# Nutritious Supplemental Foods for Pregnant Women from Food Insecure Settings: Types, Nutritional Composition, and Relationships to Health Outcomes

**DOI:** 10.1016/j.cdnut.2023.100094

**Published:** 2023-04-28

**Authors:** Mihaela A. Ciulei, Emily R. Smith, Nandita Perumal, Chioniso S. Jakazi, Christopher R. Sudfeld, Alison D. Gernand

**Affiliations:** 1 Department of Nutritional Sciences, the Pennsylvania State University, University Park, PA, United States; 2 Department of Global Health, George Washington University Milken Institute School of Public Health, Washington, DC, United States; 3 Department of Exercise and Nutrition Sciences, George Washington University Milken Institute School of Public Health, Washington, DC, United States; 4 Department of Global Health and Population, Harvard School of Public Health, Boston, MA, United States; 5 Department of Nutrition, Harvard School of Public Health, Boston, MA, United States

**Keywords:** balanced energy-protein, BEP, lipid-based nutrient supplement, LNS, women, pregnancy, birth outcomes, maternal outcomes, narrative review

## Abstract

There is growing evidence that the provision of nutritious supplemental foods to undernourished pregnant women can improve maternal and infant outcomes. However, comparing and synthesizing the evidence base is complicated by differences in interventions and products and the use of ambiguous terminology. We aimed to define 2 common types of nutritious supplemental foods used in pregnancy, balanced energy-protein (BEP) supplements and lipid-based nutrient supplements (LNS), and to review the evidence supporting each via a narrative review of systematic reviews and meta-analyses (SRMAs). Information about the nutritional composition of the food supplements and their effects on maternal and infant outcomes was abstracted. Five SRMAs (*n* = 20 trials) evaluated the effect of BEP compared with no BEP/control (comparison group commonly received iron and folic acid [IFA]). BEP foods/products ranged in calories (118–1017 kcals), protein (3–50 g), fat (6–57 g), and micronutrient content. Overall, maternal BEP improved birth weight and reduced the risk of stillbirth and small for gestational age when compared with no BEP/control in pregnancy. Three SRMAs (*n* = 5 trials) evaluated the effect of LNS compared with IFA or multiple micronutrients (MMNs). The LNS interventions comprised small- and large-quantity LNS that ranged in calories (118–746 kcals), protein (3–21 g), fat (10–53 g), and micronutrient content. LNS compared with IFA increased pregnancy duration, birth weight, and birth length and reduced the risk of small for gestational age and infant stunting; however, no beneficial effect of LNS was identified when compared with MMN. Despite heterogeneity in the nutritional composition of BEP supplements, the evidence suggests that in nutritionally at-risk populations, these products may improve birth outcomes in pregnant women. The evidence is limited but promising when LNS is compared with IFA in improving maternal and infant outcomes. Overall, BEP, compared with MMN or LNS, are key areas that have not been studied and deserve attention.

## Introduction

**FIGURE 1:**
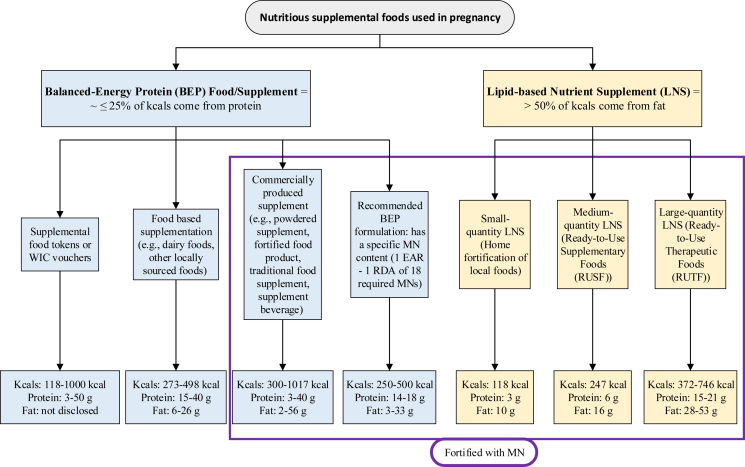
Nutritious supplemental foods used in pregnancy. Aside from 2 studies [34,38] that provided <250 kcals in BEP food/product, the total energy per day for medium-quantity and large-quantity LNS overlap with most of the BEP foods/products given in research trials; LNS products overlap in energy from protein with BEP; BEP food/products should provide <50% of energy from fats. BEP, balanced energy-protein; EAR, estimated average requirement; LNS, lipid-based nutrient supplement; MN, micronutrient; WIC, Special Supplemental Nutrition Program for Women, Infants, and Children. Small-quantity-LNS [13,44,45,55], medium-quantity-LNS [13,47], large-quantity-LNS [31,46,13]; food-based supplementation [[20], [21], [22], [23],^25^], commercially produced supplement [[26], [27], [28], [29], [30], [31], [32], [33],[35], [36], [37]], supplemental food tokens or vouchers [38,39], recommended BEP formulation [10].

Pregnancy is a unique nutritional physiologic stage characterized by an increased demand for energy, micronutrients, essential amino acids, and fatty acids that are needed for the development of new tissues, such as the fetus, and existing tissues, such as the uterus [1,2]. Poor nutrition in pregnancy is an important contributor to adverse maternal and infant outcomes [3,4]. Women with underweight (BMI <18.5 kg/m^2^) prior to pregnancy, reflecting undernutrition, are at risk of pregnancy complications, including intrauterine growth restriction and other adverse perinatal outcomes [5]. Further, inadequate gestational weight gain during pregnancy and micronutrient deficiencies also put women at risk of having preterm birth and low-birth weight infants [6]. To improve pregnant or lactating women’s nutritional status and infant outcomes in nutritionally at-risk populations, products and interventions with varying compositions of macronutrients and micronutrients have been developed; they are broadly referred to by the nutrition community under a collective term, nutritious supplemental foods (NSFs), and are shown in Figure 1.

There are 2 common types of NSFs used in pregnancy, balanced energy-protein (BEP) supplements and lipid-based nutrient supplements (LNS). In 2016, the WHO recommended that BEP supplementation be provided to pregnant women in populations with a prevalence of underweight (BMI <18.5 kg/m^2^) ≥20% [7]. Currently, there is no such recommendation regarding the use of LNS in pregnancy. These products are an important public health intervention to ensure a positive pregnancy experience for women at risk of undernourishment. As the evidence showing the benefits of these products has grown, new products and related research have emerged. Along with these advances has also come some confusion around product-related terminology and difficulty in synthesizing and comparing the evidence base. This narrative review aimed to define the nutritional composition of NSFs, such as BEP and LNS, used in pregnancy and review the evidence for these foods/products’ impact on pregnancy outcomes.

## Types of NSFs

A report published in 1998 described 3 types of nutrition interventions tested in pregnant women residing in food-insecure settings who were at risk of protein deficiency [8]. These interventions included BEP supplementation (protein accounts for <25% of the total energy content), isocaloric balanced protein supplementation (the protein in the intervention group replaces an equal quantity of nonprotein energy in the comparison group; both groups provide a similar amount of energy), and high-protein supplementation (the protein provides ≥25% of the total energy content). Based on the available evidence at that time coming from 1 trial, isocaloric balanced protein supplementation and high-protein supplementation interventions increased the risk of having small for gestational age (SGA) infants, including among undernourished pregnant women [8]. However, based on a more recent review [9], isocaloric balanced protein supplementation (based on 2 trials) had no effect on maternal and child outcomes, whereas the evidence on high-protein supplementation (based on 1 trial) indicated an increased risk of SGA. In contrast, BEP supplements reduced the incidence of intrauterine growth restriction, as measured by mean birth weight and SGA [8]. Since then, multiple forms and types of BEP supplements have been formulated and tested in pregnant women (Figure 1). There has been wide variability in the amount of energy (118–1017 kcals), protein (3–50 g), and fat (6–57 g) provided by BEP foods/products. Given the heterogeneity in products and foods administered in research trials, an Expert Consultation Report published in 2017 proposed guidelines for the macronutrient and micronutrient composition of BEP products [10]. The report recommends the following ranges: 250–500 kcals, 14–18 g protein, and 3–33 g fat. It was also recommended that the BEP supplement contain ≤25% of energy coming from protein. The specifications for micronutrients follow the United States Institute of Medicine Dietary Reference Intake guidelines [11], in which the Estimated Average Requirement was set as the minimum, and the Recommended Dietary Allowance was set as the maximum recommended in case there is concomitant iron and folic acid (IFA) or multiple micronutrients (MMNs) supplementation provided with this BEP product [10]. The recommended required micronutrients are vitamins A, D, E, K, B1, B2, B3, B6, B12, and C, iron, zinc, iodine, calcium, phosphorus, copper, and selenium, and the recommended optional micronutrients are vitamin B5, manganese, potassium, biotin, and choline [10].

Another class of nutritional products given to pregnant women is LNS, which provides the majority of energy from lipids (>50%, including essential fatty acids) but also includes protein, carbohydrates, and micronutrients [12,13]. In prior studies, LNS supplements contained soybean oil, dried skim milk, peanut, sugar, vitamin/mineral premix, maltodextrin, stabilizers, and emulsifiers [13]. Traditionally, LNS was designed to prevent wasting or stunting in children, but formulations for pregnant and lactating women have been developed to prevent maternal malnutrition [14]. To date, 3 LNS formulations (small-, medium-, and large-quantity) for pregnant women have been defined by Arimond et al. [13] (Figure 1). Small-quantity LNS provides 20 g of food per day, including 3 g (9%) of protein and 10 g (73%) of fat, and is meant to complement food in the diet. The medium-quantity LNS, which is also known as ready-to-use supplementary food, has traditionally been used to treat (or sometimes prevent) moderate acute malnutrition in children [13]. The medium-quantity LNS is between 45 and 90 g/d of supplementary food, of which 6 g (10%) is protein and 16 g (58%) is fat. Last, the large-quantity LNS, which is also referred to as ready-to-use therapeutic food, has been used to treat severe acute malnutrition in children. It provides between 180 and 280 g/d of supplementary food, of which 15 g (16%) is protein and 28 g (67%) is fat [13]. Notably, the energy provided by medium- and large-quantity LNS overlaps with some BEP products and foods, although the proportion of fat and protein differentiates the 2 types (Figure 1).

Considering the macronutrient recommendations for BEP foods or supplements, they closely follow the recommended macronutrient content of a healthy diet in adults, where 45%–65% are carbohydrates, 10%–35% are protein, and 20%–35% are fat. Thus, unlike LNS supplements, BEP products should provide <50% of energy from fat, which is an important distinction.

## Methods

### Literature search

For this narrative review, we identified systematic review and meta-analysis (SRMA) studies, including Cochrane and non-Cochrane Reviews, by searching articles in the Cochrane Database of Systematic Reviews and MEDLINE (via PubMed) databases. The search was limited to studies published in English from inception until December 2022. In our search strategy, we used the following terms for BEP interventions “balanced energy protein” OR “balanced energy protein supplement” OR “balanced energy protein supplementation” OR “balanced protein energy” OR “balanced protein energy supplement” OR “balanced protein energy supplementation” and the following terms for LNS interventions, “lipid-based nutrient supplements” OR “lipid-based nutrient supplementation.” Additionally, to identify ongoing and recently concluded intervention trials comparing BEP to the standard of care (including IFA supplements) or LNS compared with IFA or MMN administered during pregnancy, we searched clinicaltrials.gov, the International Clinical Trials Registry Platform, and MEDLINE (via PubMed) using the same aforementioned keywords.

To guide this review, our inclusion criteria included only SRMAs where BEP (i.e., food) supplementation or LNS were given to pregnant women. The comparator group in the BEP studies was left as the authors defined it, but for LNS studies, we restricted it to IFA and/or MMN. The BEP outcomes that this review focused on are low birth weight, birth weight, length, and head circumference, SGA, preterm birth, stillbirth, gestational weight gain, gestational age, neonatal mortality, and preeclampsia. For LNS, we selected the same BEP outcomes (except for gestational age) plus the following ones, birth head circumference *z*-score, maternal anemia, mortality, miscarriage, newborn MUAC, underweight, and stunting, perinatal mortality.

### Data collection

For this narrative review, we extracted data from the individual trials that were included in each SRMA study. These data include study sample size, location, study design, participant eligibility criteria, definition of undernourishment, description of control and intervention, their macronutrient and micronutrient content, and outcome estimates.

## Review of the evidence

### Nutritional composition of NSFs

#### BEP

We identified 5 SRMA studies that met our inclusion/exclusion criteria [9,[15], [16], [17], [18]]; these reviews included a total of 20 trials (Supplementary Table 1). Notably, there are 2 relevant studies conducted by Lassi et al. [17,19], but we included only the most recent one [17] because it focused on the same studies and outcomes that we are interested in as the old one. Next, the study by Perumal et al. [18] was a landscape review and not a systematic review. However, we decided to include this review because it focused on studies that included undernourished participants from Ota et al. [9] SRMA. In terms of BEP interventions, there was considerable heterogeneity in the form and nutritional composition of BEP used across trials (Supplementary Table 2). For instance, BEP encompassed dairy foods, such as milk or cheese [[20], [21], [22], [23], [24], [25]], powdered supplements [[24], [25], [26], [27], [28], [29]], fortified biscuits [[27], [28], [29]], locally sourced, traditional food supplements/foods [[30], [31], [32], [33], [34], [35], [36]], supplemental beverages[21,24,37], and supplemental food tokens or food vouchers [38,39]. The total energy content of BEP intervention in the trials varied from 118 kcal [34] to 1017 kcal [27]. Two trials [34,38] provided <250 kcals, of which 1 study [34] was later identified to provide LNS and not BEP intervention. Next, most supplements were between 250 and 500 kcal (14/18 studies; [[20], [21], [22], [23], [24], [25], [26],[28], [29], [30], [31], [32],^36^,^37^]), and 4/18 trials [27,33,35,39] provided >500 kcal total energy (Figure 1; Supplementary Table 2). For studies that provided tokens or vouchers for additional foods [38,39], the total energy content of BEP consumed between participants was challenging to approximate because of participant food choice and assessment of foods consumed. Because of the differences in the composition of BEP, the macronutrient content used in contemporary trials has also varied considerably (Figure 1; Supplementary Table 2).

The most consistently reported macronutrient in BEPs was the amount of protein, which ranged between a low of 3 g [26] to a high of 40–50 g [21,35,39]. Carbohydrate and fat content of BEPs were not consistently reported across all studies, although available evidence suggests considerable variability in these macronutrients as well. For example, among studies that provide more detailed information on the nutritional composition of BEP, fat content ranged from 6 g [20] to 57 g [27]. Last, few studies reported micronutrient composition in detail (Supplementary Table 3). Among trials that reported these data, they were primarily conducted in high-income countries [20,21,23,37]. However, other trials [27,28] used BEP interventions that had only a few micronutrients included, most commonly iron, folic acid, and calcium (Supplementary Table 3). Vitamin A, C, B1, B2, B3, B6, folic acid, B12, iron, and calcium were the most included micronutrients in the BEP supplements, whereas biotin, choline, and selenium content were rarely reported.

The control group for these BEP trials also varied, but iron (with or without other micronutrients) was given or was part of antenatal care [21,[23], [24], [25],[27], [28], [29], [30], [31],^34^,^37^]. Of these, Ceesay et al. [27] gave iron only to pregnant women with anemia. In other studies, antenatal care was specified but not the micronutrient composition [20,26,33,35]. Of these, Mora et al. [33] indicated that health care and not specifically antenatal care was available. Last, the remaining studies did not mention if a micronutrient was given or antenatal care availability [22,36,38,39]. Notably, of the studies that did not mention micronutrient composition nor antenatal care availability, the majority of them were conducted in high-income countries [20,22,26,38,39] where standard antenatal care would likely include IFA treatment (Supplementary Table 2).

#### LNS

The evidence for LNS in pregnancy is limited to 3 SRMA studies [[40], [41], [42]] that met our inclusion criteria and included 5 LNS trials. Three trials used small-quantity LNS [[43], [44], [45]] and 2 trials used large-quantity LNS [31,46]. The products differed such that in a study by Huybregts et al. [31], the large-quantity LNS was called a fortified food supplement and was in the form of a fortified spread consisting of 33% peanut butter, 32% soy flour, 15% vegetable oil, 20% sugar, and MMN. However, in these trials [[43], [44], [45]], participants were instructed to consume 1 small-quantity LNS sachet mixed with any food each day. Moore et al. [46] provided participants with large-quantity LNS in a ready-to-use package. For total calories, the 3 randomized controlled trials (RCTs) [[43], [44], [45]] that used small-quantity LNS had a standard energy content of 118 kcal/d. However, large-quantity LNS had a fairly wide difference in calories, 372 and 746 kcals/d, for the 2 studies included [31,46] (Supplementary Table 2).

The interventions used in LNS studies were generally more homogenous when compared with BEP interventions with respect to macronutrients and micronutrients, and LNS is typically compared with IFA or MMN. Regarding macronutrient content, the 3 RCTs that used small-quantity LNS [[43], [44], [45]] similarly provided 3 g (9%) of protein and 10 g (76%) of fat. The large-quantity LNS studies also used a similar percentage of calories coming from protein (21 g [11%] compared with 15 g [16%]) and fat (53 g [64%] compared with 28 g [67%]) (Supplementary Table 1; [31,46]). All 5 study interventions match the definitions of calories that should come from protein and fat for small- and large-quantity LNS described by Arimond et al. [13]. Regarding the micronutrient content in the LNS products, the 3 RCTs [[43], [44], [45]] provided the same type of micronutrients, but the composition was modified from the MMN developed by the United Nations International Multiple Micronutrient Antenatal Preparation to account for both pregnant and lactating women’s needs in food insecure settings [13]. For instance, this modified MMN-fortified LNS contained twice the amount used in previous prenatal MMN trials for B vitamins, vitamin D, E, zinc, copper, and selenium, plus another 4 micronutrients: calcium, phosphorus, potassium, and magnesium (Supplementary Table 3). However, in these trials [31,46], the MMN-fortified LNS had the micronutrient content match the 1 developed by United Nations International Multiple Micronutrient Antenatal Preparation (Supplementary Tables 2 and 3). Notably, in Moore et al. [46], aside from 2 control groups that received IFA and MMN, there were 2 large-quantity LNS groups. One group received large-quantity LNS and IFA, and the other 1 received large-quantity LNS fortified with MMN, which was 2 times the Recommended Dietary Allowance, except for IFA, which remained the same amount as the IFA group.

Two particular publications, Huybregts et al. [31] and Oaks et al. [34] (abstract only was used in published reviews), were included in reviews of BEP as well as LNS. Huybregts et al. [31] labeled the product as a fortified food supplement and not as BEP or LNS. Nevertheless, based on the LNS definition that requires the majority of energy to come from lipids (>50%), this intervention falls under large-quantity LNS because it provides 67% of kcals from fat (28 g). However, the total amount of energy (372 kcal) and protein (27 g, 16%) overlap with the BEP category. With respect to Oaks et al. [34], the authors labeled their supplement as LNS and indicated that it contains 118 kcals, 22 micronutrients, and protein. Thus, because of limited information in the abstract, it was wrongly included in a review focusing on BEP trials. When the full study was published [43], the authors defined the product as small-quantity LNS, which follows the LNS definition given that 76% of kcals come from fat (10 g), and it was included in all reviews focusing on LNS trials (Supplementary Table 1).

### Effect of NSFs on maternal and infant outcomes

#### BEP

As previously discussed, the existing evidence on BEP supplementation compared with no BEP/control given during pregnancy comes from 5 SRMAs [9,[15], [16], [17], [18]], and the results are indicated in Table 1.

**TABLE 1:** Summary estimates from systematic reviews and meta-analyses that tested the effect of balanced energy-protein (BEP) compared with no BEP/control^1^ in pregnant women on maternal and infant outcomes

Outcomes	Imdad and Bhutta, 2012	Ota, 2015	Stevens, 2015	Lassi, 2021	Perumal, 2021
		Estimate (95% CI), number of studies (*n*)			
LBW	RR = 0.68 (0.51, 0.92); *n* = 5^2^	-	-	RR = 0.60 (0.41, 0.86); *n* = 3	RR = 0.83 (0.61, 1.12); *n* = 4^2^
Birth weight (g)	d = 73.8 (30.4, 117.2); *n* = 16^2^	d = 40.96 (4.66, 77.26); *n* = 11	d = 0.20 (0.03, 0.38); *n* = 7^2^	d = 107.28 (68.51, 146.06); *n* = 3	-
Birth length (cm)	d = 0.16 (0.02, 0.31); *n* = 7	d = 0.18 (–0.04, 0.40); *n* = 5	d = 0.23 (–0.04, 0.50); *n* = 5^2^	d = 0.28 (–0.36, 0.92); *n* = 2	-
SGA	RR = 0.66 (0.49, 0.89); *n* = 9^2^	RR = 0.79 (0.69, 0.90); *n* = 7	-	RR = 0.71 (0.54, 0.94); *n* = 5	-
Preterm birth	RR = 0.96 (0.80, 1.15); *n* = 6	RR = 0.96 (0.80, 1.16); *n* = 5	-	RR = 0.86 (0.50, 1.46); *n* = 2	-
Stillbirth	RR = 0.66 (0.40, 0.98); *n* = 4	RR = 0.60 (0.39, 0.94); *n* = 5^2^	-	RR = 0.39 (0.19, 0.80); *n* = 3^2^	-
GWG (g/wk)	d = 20.74 (1.46, 40.02); *n* = 10	d = 18.63 (–1.81, 39.07); *n* = 9	-	-	-
Neonatal mortality	RR = 0.67 (0.59, 0.82); *n* = 4	RR = 0.68 (0.43, 1.07); *n* = 5^2^	-	RR = 0.58 (0.32, 1.06); *n* = 1	-
Preeclampsia	RR = 1.20 (0.77, 1.89); *n* = 3	RR = 1.48 (0.82, 2.66); *n* = 2	-	-	-
Gestational age (wk)	-	d = –0.10 (–0.22, 0.01); *n* = 6	-	-	-
Birth head circumference (cm)	d = 0.07 (–0.02, 0.16); *n* = 7	d = 0.04 (–0.08, 0.17); *n* = 5	d = 0.17 (–0.07, 0.41); *n* = 3^2^	-	-

Abbreviations: d, mean difference; GWG, gestational weight gain; SGA, small for gestational age; "-", not assessed; LBW, low birth weight.

1 The comparison group varied in what it included, but in most cases, iron with or without other micronutrients was prescribed or likely available through antenatal care; however, for some studies [22,36,38,39], there was no indication of iron prescription or antenatal availability.

2 The primary outcome.

Imdad and Bhutta’s [15] review focused on assessing the effect of BEP compared with control on pregnancy outcomes. This work identified 16 studies, of which 8 were conducted in low- and middle-income countries (Supplementary Table 1; [21,27,[29], [30], [31], [32], [33],^35^]) and 8 were conducted in high-income ones (Supplementary Table 1; [20,[22], [23], [24], [25],[37], [38], [39]]). The pooled findings for primary outcomes indicated that BEP compared with control increased birth weight (g) by 73.78 g (95% CI: 30.42, 117.15; 16 studies; Table 1), and when this assessment was conducted among undernourished pregnant women, the effect of BEP compared with control was more pronounced (mean difference [d] = 100.86, 95% CI: 56.14, 145.58) than in nourished pregnant women (d = 22.58, 95% CI: –27.17 to 73.32; data not shown). BEP, compared with control, also reduced the risk of low-birth weight by 32% (RR = 0.68, 95% CI: 0.51, 0.92; 5 studies) and the risk of SGA by 34% (RR = 0.66, 95% CI: 0.49, 0.89; 9 studies). However, these latter findings should be interpreted with caution because there was a high degree of heterogeneity, I^2^ = 80% and I^2^ = 87% for low birth weight and SGA, respectively. In terms of secondary outcomes, BEP, compared with control, significantly reduced the risk of stillbirth by 38% (RR = 0.62; 95% CI: 0.40, 0.98; 4 studies) and increased gestational weight gain by 20.74 g/wk (95% CI: 1.46, 40.02 g; 10 studies; Table 1). There was no effect on neonatal mortality, birth length, head circumference, or preeclampsia.

The Cochrane review published by Ota et al. [9] included 12 BEP studies, of which 7 were from low- and middle-income countries, including undernourished populations [26,27,30,31,33,37], and the rest (*n* = 6) included well-nourished populations, primarily from high-income countries [21,24,25,34,35,38]. The pooled findings for primary outcomes revealed that BEP compared with control, significantly reduced the risk of stillbirth by 40% (RR = 0.60; 95% CI: 0.39, 0.94; 5 studies) but did not impact neonatal mortality (Table 1). For secondary outcomes, BEP, compared with control, decreased the risk of SGA by 21% (RR = 0.79; 95%: 0.69, 0.90; 7 studies) and increased birth weight by 40.96 g (95% CI: 4.66, 77.26; 11 studies; Table 1). No other secondary outcomes (birth length and head circumference, preterm birth, gestational weight gain, preeclampsia, and gestational age) were different between groups (Table 1). Nevertheless, the stillbirth and SGA findings were largely influenced by Ceesay et al. [27] trial in Gambia, which was the only trial to find significant positive effects on either outcome independently. Further, a subgroup analysis suggested that the magnitude of the effect of supplementation on birth weight may be larger for the broadly defined subgroup of trials conducted among populations of “undernourished” pregnant women as compared with “adequately nourished” populations. However, even among the undernourished subgroup of trials, there was significant heterogeneity in the effect of BEP interventions on birth weight (I^2^ = 54%; [9]). In a subsequent study conducted by Perumal et al. [18], they included only the studies from the Ota et al. [9] review that defined undernourished pregnant women based on BMI of <18.5 kg/m^2^ (4 studies [21,26,27,31]). Perumal et al. [18] revealed that in undernourished pregnant women, BEP was not superior to control in reducing low birth weight, which was the only outcome tested (RR = 0.83; 95% CI: 0.61, 1.12; 4 studies; Table 1).

The 2 recently published SRMAs by Stevens et al. [16] and Lassi et al. [17] have results similar to Ota et al. [9]. However, unlike Imdad and Bhutta [15] and Ota et al. [9], Stevens et al. [16] restricted their review to studies from low- and middle-income countries (7 studies [21,26,27,30,31,33,36]; Supplementary Table 1) that focused on testing the effect of BEP given to undernourished pregnant women on child growth. Their pooled findings for the primary outcomes indicate that BEP, compared with control, had a moderately significant effect on birth weight (g) (d = 0.20; 95% CI: 0.03, 0.38; 7 studies) but no effect on birth length (Table 1). Similarly to Stevens et al. [16], Lassi et al. [17] study restricted the review to 8 studies from low- and middle-income countries (Supplementary Table 1; [[27], [28], [29], [30],^32^,^33^,^35^,^36^]). They focused on the effects of BEP supplementation given during preconception and pregnancy on maternal, neonatal, and child outcomes. Unlike the prior studies, the participants in this work were either healthy, undernourished, or obese without any comorbidities. The pooled results for the primary outcome, stillbirth, indicated that BEP compared with control, significantly reduced risk (RR = 0.39; 95% CI: 0.19, 0.80; 3 studies). For secondary outcomes, BEP, compared with control, significantly increased birth weight (g) (d = 107.28; 95% CI: 68.51, 146.06; 3 studies), decreased the incidence of SGA by 29% (RR = 0.71; 95% CI: 0.54, 0.94; 5 studies), and reduced low birth weight by 40% (RR = 0.60; 95% CI: 0.41, 0.86; 3 studies; Table 1). Birth length and preterm birth were not significantly different between BEP and control. Notably, the authors revealed that the results from these studies are limited by low quality.

#### LNS

Three SRMAs tested the effect of LNS compared with control (IFA or MMN) in pregnant women on maternal and child outcomes [[40], [41], [42]]. The findings from these reviews are based on 4–5 studies conducted in Africa and South Asia, which are shown in Table 2 [31,[43], [44], [45], [46]]. In all SRMAs, the small- and large-quantity LNS administered were combined.

**TABLE 2:** Summary estimates from systematic reviews and meta-analyses that tested the effect of lipid-based nutrient supplements (LNS) compared with iron and folic acid (IFA) or multiple micronutrients (MMN) in pregnant women on maternal and infant outcomes

Outcomes	Das, 2018		Oh, 2020	Keats, 2021
	LNS vs. IFA	LNS vs. MMN	LNS vs. MMN	LNS vs. MMN
	Estimate (95% CI), number of studies (*n*)			
LBW	RR = 0.87 (0.72, 1.05); *n* = 3^1^	RR = 0.92 (0.74, 1.14); *n* = 3^1^	RR = 0.92 (0.75, 1.13); *n* = 4^1^	RR = 0.92 (0.75, 1.13); *n* = 4^1^
Birth weight (g)	d = 53.28 (28.22, 78.33); *n* = 3^1^	d = 23.67 (−10.53, 57.86); *n* = 3^1^	-	-
Birth length (cm)	d = 0.24 (0.11, 0.36); *n* = 3^1^	d = 0.20 (−0.02, 0.42); *n* = 3^1^	-	-
SGA	RR = 0.94 (−0.89, 0.99); *n* = 3^1^	RR = 0.95 (0.84, 1.07); *n* = 3^1^	RR = 0.96 (0.86, 1.07); *n* = 4	RR = 0.96 (0.86, 1.07); *n* = 4;
Preterm birth	RR = 0.94 (0.80, 1.11); *n* = 3^1^	RR = 1.15 (0.93, 1.42); *n* = 3^1^	RR = 1.15 (0.93, 1.42); *n* = 4	RR = 1.15 (0.93, 1.42); *n* = 4
Stillbirth	RR = 1.14 (0.52, 2.48); *n* = 3	-	RR = 0.25 (0.08, 0.78); *n* = 2	RR = 0.47 (0.12, 1.81); *n* = 3
GWG (g/wk)	d = 0.46 kg (−0.44, 1.36); *n* = 2^1^	d = 0; *n* = 2^1^	-	-
Neonatal mortality	RR = 0.72 (0.47, 1.10); *n* = 3	RR = 0.88 (0.36, 2.15); *n* = 1	RR = 0.81 (0.45, 1.45); *n* = 3	RR = 0.81 (0.45, 1.45); *n* = 3
Gestational age (wk)	d = 0.18 (0.04, 0.32); *n* = 3	d = −0.07 (−0.26, 0.12); *n* = 3	-	-
Birth head circumference (cm)	d = 0.20 (0.20, 0.20); *n* = 2	d = 0.08 (−0.16, 0.31); *n* = 2	-	-
Maternal anemia	RR = 2.35 (1.67, 3.30); *n* = 1^1^	RR = 1.40 (1.07, 1.82); *n* = 1^1^	-	-
Maternal mortality	RR = 0.53 (0.12, 2.41); *n* = 3^1^	-	-	-
Miscarriage	RR = 0.87 (0.66, 1.14); *n* = 2	-	RR = 1.12 (0.69, 1.80); *n* = 3	RR = 1.12 (0.69, 1.80); *n* = 3
Newborn MUAC (cm)	d = 0.12 (−0.02, 0.26); *n* = 2	d = 0.07 (−0.01, 0.16); *n* = 2	-	-
Newborn underweight	RR = 0.84 (0.63, 1.13); *n* = 2	RR = 0.78 (0.46, 1.33); *n* = 1	-	-
Newborn stunting	RR = 0.82 (0.71, 0.94); *n* = 2	RR = 1.06 (0.75, 1.51); *n* = 1	-	-
Birth head circumference *z*-score	d = 0.11 (0.04, 0.18); *n* = 3	d = 0.10 (−0.01, 0.21); *n* = 2	-	-
Perinatal mortality (≤7d)	-	-	RR = 1.01 (0.65, 1.65); *n* = 3^1^	RR = 1.01 (0.65, 1.65); *n* = 3

Abbreviations: d, mean difference; GWG, gestational weight gain; IFA, iron and folic acid; LNS, lipid-based nutrient supplements; MMN, multiple micronutrients; SGA, small for gestational age; “-” not assessed; LBW, low birth weight.

1 The primary outcome.

The Cochrane SRMA published by Das et al. [40] in 2018 included 4 studies, all from low- and middle-income countries [31,[43], [44], [45]]. However, the study conducted by Huybregts et al. [31] was not included because of the high risk of attrition bias (>20% attrition). The pooled findings on primary outcomes indicated that LNS was not superior to IFA in improving gestational weight gain (kg) per week, maternal mortality, low birth weight, or the rate of preterm births (Table 2). However, LNS improved birth weight (g) (d = 53.28; 95% CI: 28.22, 78.33; 3 studies), birth length (cm) (d = 0.24; 95% CI: 0.11, 0.36; 3 studies), and lowered the risk of SGA (RR = 0.94; 95% CI: 0.89, 0.99; 3 studies; Table 2). For secondary outcomes, women who consumed LNS compared with IFA showed an increase in gestational age (weeks) (d = 0.18; 95% CI: 0.04, 0.32; 3 studies) and an increase in risk of maternal anemia by 2-fold (RR = 2.35; 95% CI: 1.67, 3.30; 1 study). However, there was no difference between groups for stillbirths, miscarriages, head circumference (except for a higher *z*-score in LNS compared with IFA [d = 0.11; 95% CI: 0.04, 0.18; 3 studies]), and MUAC. The prevalence of newborn stunting was 18% lower in the LNS than in the IFA group (RR = 0.82; 95% CI: 0.71, 0.94; 2 studies), but no group differences were observed for underweight or neonatal deaths (Table 2). When LNS was compared with MMN, there was no clear advantage between groups for maternal gestational weight gain (kg), newborn underweight, stunting, and mortality; 1 study. However, LNS rather than MMN increased the risk of maternal anemia (RR = 1.40; 95% CI: 1.07, 1.82; 1 study). Next, the effect sizes for low birth weight, birth weight (g), birth length (cm), SGA, and preterm births also indicated that LNS and MMN might be comparable (Table 2). Of note, these findings should be interpreted with caution as the community-based Bangladesh trial [45] had the largest sample size (*n* = 4011) when compared with the other trials; as such, it heavily weighted most outcomes (63%–65%).

Recently, 2 SRMA studies in LNS in pregnancy were published by Oh et al. [41] in 2020 and Keats et al. [42] in 2021. These 2 reviews compared LNS with MMN and included the same 4 trials [31,43,44,46], which were conducted in low- and middle-income countries. Also, 3 of the trials included in the reviews [31,43,44,46] were conducted in rural settings, with the exception of the study by Adu-Afarwuah et al. [43], which was conducted in a peri-urban area. Two trials provided small-quantity LNS [44], and 2 provided large-quantity LNS [31,46], but in both meta-analyses, these interventions were combined. Oh et al. [41] focused on which antenatal supplementation interventions are effective at improving maternal and child health, nutrition, and mortality outcomes and conducted subgroup analyses. The work by Keats et al. [42] focused on MMN supplementation and assessed the effect of LNS compared with MMN [42]. When evaluating the effect of LNS across all 4 trials against MMN, the same primary and secondary outcomes were analyzed by Oh et al. [41] and Keats et al. [42] (Table 2). Overall, in both reviews conducted, LNS was not superior to MMN in lowering the risk of low-birth weight or perinatal mortality (Table 2). There was also no difference in the secondary outcomes of neonatal mortality, preterm birth, stillbirth, miscarriage, and SGA between LNS and MMN. Of note, in sensitivity analyses, both reviews found, after the removal of 1 study [31] because of the high risk of bias, that stillbirth was significantly lower in the LNS compared with the MMN group (RR = 0.25; 95% CI: 0.08, 0.78; 2 studies). In the original analysis, there was no effect of LNS supplementation (RR = 0.47; 95% CI: 0.12, 1.81; 3 studies; [Table 2]).

### Challenges interpreting evidence

There is substantial heterogeneity in the population and comparison groups in the existing BEP trials, which complicates the interpretation of the existing evidence. For instance, approximately half of BEP trials were conducted in high-income countries. Although most studies evaluated the efficacy of BEP using an RCT design, a few studies were quasi-experimental [20,23], pre-intervention/post-intervention design [29,38,39], or sequential-randomized [32]; Supplementary Table 1). Heterogeneity in study design and settings has also led to important differences in evidence synthesis efforts for BEP (Supplementary Table 1) such that the 5 recent SRMAs that studied the effect of BEP intervention on perinatal outcomes have included a different set of studies (Supplementary Table 1). In regards to LNS studies, the majority were RCTs and were all conducted in low- and middle-income countries (Supplementary Table 1; [31,[43], [44], [45], [46]]. However, Huybregts et al. [31] study was a non-blinded RCT, and Mridha et al. [45] study was a cluster-randomized effectiveness trial.

Importantly, although most BEP studies were conducted among “undernourished” pregnant women, there was heterogeneity in the criteria used to define undernourished status (Supplementary Tables 1 and 2). For example, Blackwell [21] defined participants as being at risk of undernutrition if they were of low-socioeconomic status (defined by the lack of electric appliances in the home) and consumed a “marginal diet” consisting of a daily energy intake of ∼2,000 kcal/d with <40 g/d of protein. A few studies used low-socioeconomic status (e.g., based on family income [23,30,39]) or residence in a poor neighborhood or slum area [33,37] as criteria to identify women at risk of poor nutrition during pregnancy. Most BEP studies defined participants as being undernourished based on maternal nutritional status, although the metric used to assess nutritional status differed: low maternal triceps skinfold thickness, low maternal weight, low maternal height, poor weight gain in early pregnancy, or low BMI at the start of pregnancy (Supplementary Table 2). Two studies conducted in Gambia [27,29] used seasonality as the defining criteria for nutritional vulnerability, given that dwindling food supply and heavy agricultural work from June to October mark the “hungry” season. Some studies, however, were conducted among adequately nourished pregnant women [24,25,32], whereas others did not specifically consider the nutritional status of participants as eligibility criteria for enrollment [31,[34], [35], [36],^38^].

Similarly, the LNS study populations varied greatly in geographic contexts (different countries or regions) and in the types of the target population [[40], [41], [42]]. There were no unified criteria defined for “undernourishment,” and in most studies, settings were chosen on the basis of being of low-socioeconomic status and/or rural [45,46], having a high prevalence of macronutrient and micronutrient deficiencies [31], and of low birth weight [43]. Overall, the heterogeneity in the participant inclusion criteria and definitions used to discern “undernourishment” across studies of LNS reflect the importance of considering context-specificity in LNS provision.

There was considerable heterogeneity in the comparison groups used across BEP studies. For instance, the control group for 11 of 20 studies indicated iron prescription, 3 of 20 studies indicated antenatal care availability, and 4 of 20 studies did not provide information on iron prescription or antenatal care availability. The available SRMA studies have compared BEP supplementation with no BEP/control, which mainly included standard of care, such as IFA, but should also compare it with MMN. In contrast, LNS studies commonly compare LNS with IFA or MMN supplements because they share the vitamin and mineral components with varying compositions (Supplementary Table 3; [40]). The differences in comparison groups for BEP compared with LNS studies make interpreting the results challenging. However, the outcomes evaluated across studies that included either BEP or LNS were relatively consistent and are shown in Supplementary Table 4.

### Ongoing or recently completed studies

We identified 5 ongoing and recently concluded intervention trials comparing BEP to the standard of care (including IFA supplements) and 1 trial that compared LNS with IFA or MMN administered during pregnancy. Notably, BEP interventions are also being tested in preconception and lactation, as the evidence is important but lacking. The pregnancy BEP studies that we identified are in Ethiopia (Enhancing Nutrition and Antenatal Infection Treatment for Maternal and Child Health [ENAT]), Nepal (Mothers and Infants Nutrition Trial [MINT]), Burkina Faso (MIcronutriments pour la SAnté de la Mère et de l’Enfant [MISAME-III]), Pakistan (Mumta Pregnant Women Trial [MumtaPW]), and India (Women and Infants Integrated Interventions for Growth Study [WINGS]; Table 3). These BEP studies are similar in sample sizes, between 1776 and 2400. The recently published RCT study in Niger that tested LNS compared with IFA or MMN has a sample size of 3332 [47].

**TABLE 3:** Ongoing or recently completed intervention trials of balanced energy-protein and lipid-based nutrient supplements

Trial abbreviation, country	Population	Nutrition intervention	Comparison	Intervention nutritional composition			MMN composition
				Calories	Protein	Fat	
MISAME-III, Burkina Faso^1^	Pregnant women 15–40 y; gestational age <20 wk (*n* = 1776)	BEP (PlumpyMom) fortified with MMN^7^ during:	Standard of care (including IFA)	393 kcals	14.5 g	26 g	Vitamins A, C, D, E, K, B1, B2, B6, and B12, folic acid, niacin, calcium, selenium, zinc, iron, iodine, copper, and phosphorus
		A: Pregnancy					
		B: Lactation					
		C: Pregnancy and lactation					
MINT, Nepal^2^	Pregnant women 15–30 y; (*n* = 1800)	BEP (PlumpyMom) fortified with MMN^7^ during:	Standard of care (including IFA)	400 kcals	14.5 g	25.9 g	Vitamins A, C, D, E, K, B1, B2, B6, and B12, folic acid, niacin, calcium, selenium, zinc, iron, iodine, copper, and phosphorus
		A: Pregnancy					
		B: Lactation					
		C: Pregnancy and lactation					
MumtaPW, Pakistan^3^	Pregnant women 13–49 y; gestational age >8 wk and <19 wk; (*n* = 1884)	A: Ready-to-use-supplementary food (RUFS)/(BEP) fortified with MMN	Standard of care (including IFA)	400 kcals	10.5 g	Not indicated	Vitamins A, C, E, B1, B2, B6, and B12, niacin, pantothenic acid, folic acid, calcium, magnesium, selenium, zinc, iron, iodine, copper, phosphorus, potassium, and manganese
		B: BEP + 2000 mg Azithromycin (20 and 28 wk of gestation)					
		C: BEP + 450 mg Choline and 100 mg Nicotinamide (from 20 wk of gestation)					
ENAT, Ethiopia^4^	Pregnant women age; gestational age <24 wk; (*n* = 2400)	Nutrition education/counseling, IFA, iodized salt, BEP (corn soya blend (SuperCereal) fortified with MMN only to pregnant women with MUAC <23 cm) + infection management	Standard of care (including IFA) + infection management	MUAC <23 cm: 760 kcals	MUAC <23 cm: 28 g	Not indicated	Vitamins A, C, D, E, B2, B3, B6, and B12, calcium, and phosphorus
WINGS, India^5^	Preconception, pregnancy, lactation 18–30 y; (*n* = 2400)	A: Pre- and peri-conception: screen and treat malnutrition and anemia, provide IFA, multiple micronutrients, locally prepared snacks, eggs, or milk	Standard of care	BMI 16–18.5 kg/m^2^: 500 kcal; BMI <16 kg/m^2^: 1000 kcal	BMI 16–18.5 kg/m^2^: 6–10 g; BMI <16 kg/m^2^: 12–20 g	Not indicated	Vitamins A, C, B12, B6, B1, B2, zinc, selenium, copper, magnesium, and iodine
		B: Pregnancy: provide IFA, MMN, locally prepared snacks and milk, and monitor weight	Routine antenatal care	BMI <25 kg/m^2^: second trimester, 280 kcal and third trimester, 470 kcal; BMI <18.5 kg/m^2^: second and third trimester, 500 additional kcal	BMI <25 kg/m^2^: second trimester, 8 g and third trimester, 27; BMI <18.5 kg/m^2^: second and third trimester, 20 additional g		Vitamins A, C, B1, B2, B6, and B12, zinc, selenium, copper, magnesium, and iodine
		C: Mothers (0–24 mo): 0–6 mo: IFA, calcium, vitamin D, MMN, locally prepared snacks, milk supplementation, lactation support for early and exclusive breastfeeding; 6–24 mo: promote timely complementary feeding and continued breastfeeding, provide quality food, monitor inadequate weight gain	Routine postnatal care and early childhood care	570 kcal	21 g		0–6 mo: vitamin A, C, B1, B2, B6, and B12, zinc, selenium, copper, magnesium, and iodine
Niger^6^	Pregnant women; gestational age <30 wk; (*n* = 3332)	LNS (medium-quantity) fortified with MMN^8^ during: pregnancy	IFA or MMN	237 kcal	5.2 g	20 g	Vitamins A, C, D, E, J, B1, B2, B3, B5, B6, and B12, folic acid, calcium, phosphorus, potassium, magnesium, zinc, copper, iron, manganese, iodine, and selenium

Abbreviations: BEP, balanced energy-protein; BMI, body mass index; ENAT, Enhancing Nutrition and Antenatal Infection Treatment for Maternal and Child Health; IFA, iron and folic acid; LNS, lipid-based nutrient supplements; MINT, Mothers and Infants Nutrition Trial; MISAME III, MIcronutriments pour la SAnté de la Mère et de l’Enfant; MMN, multiple micronutrients; MumtaPW, Mumta Pregnant Women Trial; WINGS, Women and Infants Integrated Interventions for Growth Study.

1 https://clinicaltrials.gov/ct2/show/NCT03533712.

2 https://clinicaltrials.gov/ct2/show/NCT03668977.

3 https://clinicaltrials.gov/ct2/show/NCT04012177.

4 https://clinicaltrials.gov/ct2/show/NCT03533712.

5 https://trialsearch.who.int/?trialid=CTRI/2017/06/008908.

6 https://clinicaltrials.gov/ct2/show/NCT02145000?term=NCT02145000.

7 Supplement follows the recommendations of the Expert Consultation Report [8].

8 The MMN in fortified LNS and in tablets contains double the recommended dietary allowance for each micronutrient.

The MISAME-III and MINT trials use a factorial design to assess the impact of a fortified BEP (produced by the Nutriset company) in pregnancy alone, in lactation alone, and in pregnancy and lactation together [[48], [49], [50]]. The ENAT trial is testing the effect of IFA, iodized salt, enriched Corn Soya Blend type of fortified BEP (a World Food Program product called Super Cereal given only to women with MUAC of <23 cm), and infection management to pregnant women only [51]. The MumtaPW trial investigates the effect of fortified BEP (a World Food Program product called Mumta that provides high-energy biscuits) in combination with infection prophylaxis (2000 mg of Azithromycin in infants) or a choline and nicotinamide (i.e., niacin) supplement given to pregnant women only [52]. In contrast, the WINGS trial provides locally prepared snacks and/or foods along with tablets of IFA and MMN to women during pre- and peri-conception, pregnancy, and postpartum (0–6 mo), based on BMI status (Table 3). In pregnancy, for instance, it provides locally prepared snacks for women with a BMI of <25 kg/m^2^ that contains 210 kcal and 12 g protein in the second trimester and 400 kcal and 21 g protein in the third trimester [53]. Additionally, throughout pregnancy, all women receive milk (180 mL, 70 kcal, and 6 g of protein) for 6 d a week, and underweight women (BMI of <18.5 kg/m^2^) receive a hot home-cooked meal for breakfast that contains 500 kcal and 20 g of protein for 6 d a week [53]. Thus, depending on the participant’s nutritional status, she will receive 280–780 kcals and 8–28 g (14%) of protein in the second trimester and 470–970 kcals and 27–47 g (19%) of protein in the third trimester (Table 3). Last, all trials will assess birth outcomes, including birth weight, preterm birth, and SGA, and some will assess infant health and growth over the first 6 mo of life (Supplementary Table 4).

Regarding the micronutrient content included in these trials, MISAME-III and MINT’s product follows most closely the micronutrient formulations recommended by the 2017 Expert Consultation Report [10]. The fortified BEP in the ENAT trial contains 10 of 18 required micronutrients recommended by the report [10] but also provides IFA and iodized salt. The MumtaPW trial’s fortified BEP product provides 15 of 18 required micronutrients but also provides 4 additional micronutrients (magnesium, manganese, potassium, and vitamin B5) included in the optional list by the report [10]. Of note, a separate intervention arm receives choline (optional micronutrient) and additional niacin along with the BEP supplement. The WINGS trial provides micronutrients in the form of tablets, such as IFA and MMN, that contain 10 of 18 required micronutrients and 1 optional micronutrient (magnesium). Mothers (≤6 mo postpartum) also receive calcium and vitamin D tablets. Across all trials, the comparator was standard of care, IFA, but in MINT and WINGS, women were encouraged to use IFA available through the national antenatal care, and in ENAT, pregnant women also received infection management.

The trial from rural Niger [47] provided a MMN-fortified medium-quantity LNS or IFA or MMN with double the recommended dietaryallowance for each micronutrient. The medium-quantity LNS was a 40 g ready-to-use sachet of food and contained the same 22 micronutrients as the MMN [47]. This RCT trial has the potential to improve our understanding of the effect of medium-quantity LNS, which has not been compared with IFA or MMN in pregnant women from a setting with a high prevalence of maternal and child undernutrition.

### Future directions

Although the existing evidence on BEP supplementation indicates improved maternal and infant outcomes, the population, intervention and comparator groups, and study designs differed between the 5 SRMA studies [9,[15], [16], [17], [18]] included in this narrative review. As such, future trials should focus on pregnant women from low- and middle-income countries at risk of or undernourished, provide BEP intervention that follows the recommendations listed in the report [10], compare BEP with MMN or LNS, and ideally employ an RCT design. The 3 review studies [[40], [41], [42]] that tested LNS compared with IFA or MMN included the same 5 trials (all RCTs); as such, the evidence is more homogenous than the BEP one. Overall, LNS, which comprised of small- and large-quantity LNS, was superior to IFA but not MMN for maternal and infant outcomes. However, there is a wide range of energy and macronutrients in the LNS intervention. Thus, future trials should test medium- and large-quantity LNS in at risk of or undernourished pregnant women from low-and middle-income countries that follow the Arimond et al. [13] definitions to clarify if additional energy and macronutrients are not superior to MMN. Last, BEP and LNS trials should report the dietary intake and total calorie intake of study arms to clarify whether BEP and LNS are replacing or increasing the calories in the diet.

In conclusion, BEP and LNS are the most researched NSF interventions given to pregnant women at risk of undernourishment who predominantly reside in food-insecure settings. Given that their popularity has grown substantially, this narrative review aimed to define the BEP and LNS types, nutrition composition differences, and their effects on health outcomes via an assessment of findings in a SRMA studies. BEP supplements differ from LNS because they provide ≤25% of energy from protein, and LNS provides >50% of energy from fat (including essential fatty acids). However, when LNS is provided in medium- and large-quantity, the energy proportion overlaps with most of the BEP interventions. In addition, these products can be, and typically are, fortified with micronutrients or administered along with IFA or micronutrients in the field.

BEP interventions are diverse and can be in food-based supplementation forms, such as dairy products, commercially produced forms, such as powdered supplements, food tokens, or vouchers. In prior interventions, the foods or products included under BEP ranged in calories (118–1017 kcals), protein (3–50 g), and fat (6–57 g). Cumulatively, evidence for the use of BEP supplements comes from 5 recently published SRMA studies (*n* = 20 trials), which clearly suggest that BEP supplementation is beneficial for improving birth weight, SGA, and stillbirth outcomes when compared with no BEP/control (comparison group commonly received IFA). However, additional research is necessary to clarify the BEP supplement composition needed to achieve positive maternal and child health outcomes, which pregnant women would benefit from such intervention, and the comparison group (only half the studies indicated giving iron or iron being part of antenatal care). In response to this need, a prospective, individual participant data meta-analysis is underway and will include new ongoing or recently completed BEP trials only, which are all compared with IFA [54]. As part of this work, BEP supplementation given in lactation will also be examined (https://osf.io/9nq7z).

With respect to LNS, these products have been created in small-, medium-, and large-quantity formulations. Results from 3 SRMA studies (*n* = 5 trials) indicate that LNS is beneficial when compared with IFA but not to MMN in outcomes, such as gestational duration and infant anthropometry. However, the LNS evidence is limited and includes a wide range of energy (118–746 kcals), protein (2.6–20.8 g), and fat (10.0–52.6 g), as there is a need for more homogenous LNS evidence to clarify if the additional calories and macronutrients in LNS are not superior to the micronutrients provided in MMN in undernourished pregnant women populations.

## Funding

Supported by the Bill & Melinda Gates Foundation, investment grant number INV-022373.

## Data availability

Data described in the manuscript, code book, and analytic code will not be made available because there is no dataset; this is a narrative review of existing literature

## Appendix A Supplementary data

Multimedia component1 Multimedia component1
