# Supplementary material for: Nutritious Supplemental Foods for Pregnant Women from Food Insecure Settings: Types, Nutritional Composition, and Relationships to Health Outcomes
Source: Curr Dev Nutr. 2023 Apr 28;7(6):100094. doi: 10.1016/j.cdnut.2023.100094 (PMC10209485; doi:10.1016/j.cdnut.2023.100094)
Supplement: Multimedia component1 [file mmc1.docx]

**Supplemental Materials**

**Supplemental Table 1**: Studies included in published systematic reviews and meta-analyses of BEP and LNS trials

| **Study, year** | **Study location** | **Study design** | **Conducted in ‘undernourished’ women or stratified analyses presented?** | **Included in review?** | | | | |  |
| --- | --- | --- | --- | --- | --- | --- | --- | --- | --- |
|  |  |  |  | **Imdad 2012** | **Ota 2015** | **Stevens 2015** | **Lassi 2021** | **Perumal 2021** |  |
|  |  |  |  |  |  |  |  |  |  |
| **BEP** |  |  |  |  |  |  |  |  |  |
| Atton 1990 | England, UK | Quasi-RCT | Y | Y | N | N | N | N |  |
| Blackwell 1973 | Taiwan | RCT | Y | Y | Y | Y | N | Y |  |
| Ceesay 1997 | Gambia | Cluster- RCT | Y | Y | Y | Y | Y | Y |  |
| Campbell-Brown 1983 | Scotland, UK | RCT | Y | Y | N | N | N | N |  |
| Dwarkanath 2016 | India | RCT | Y | N | N | N | Y | N |  |
| Elwood 1981 | South Wales, UK | Pre/post design | N | Y | Y | N | N | N |  |
| Girija 1984 | India | RCT | Y | Y | Y | Y | Y | N |  |
| Huybregts 2009 | Burkina Faso | RCT | Y | Y | Y | Y | N | Y |  |
| Kardjati 1988 | Indonesia | RCT | Y | N | Y | Y | N | Y |  |
| Kaseb 2002 | Iran | Sequentially randomized intervention study | N | Y | N | N | Y | N |  |
| Mardones-Santander 1983 | Chile | Quasi-RCT | Y | Y | N | N | N | N |  |
| Metcoff 1985 | United States | Pre/post design | Y | Y | N | N | N | N |  |
| Mora 1978 | Colombia | RCT | Y | Y | Y | Y | Y | N |  |
| Oaks 2014 | Ghana | RCT | N | N | Y | N | N | N |  |
| Prentice 1987 | Gambia | Non-randomized intervention (pre/post design) | Y | Y | N | N | Y | N |  |
| Ross 1985 | South Africa | RCT | N | Y | Y | N | Y | N |  |
| Rush 1980 | United States | RCT | Y | Y | Y | N | N | N |  |
| Tontisirin 1986 | Thailand | RCT | N | N | N | Y | Y | N |  |
| Viegas 1982 a) | UK | RCT | Y | Y | Y | N | N | N |  |
| Viegas 1982 b) | UK | RCT | N | Y | Y | N | N | N |  |
| **LNS** |  |  |  | **Das et al., 2018** | **Oh et al., 2020** | **Keats et al., 2021** |  |  |  |
| Adu-Afarwuah 2015 | Ghana | RCT | N | Y | Y | Y |  |  |  |
| Ashorn 2015 | Malawi | RCT | N | Y | Y | Y |  |  |  |
| Huybregts 2009 | Burkina Faso | RCT | N | Y | Y | Y |  |  |  |
| Moore 2012 | Gambia | RCT | N | N | Y | Y |  |  |  |
| Mridha 2016 | Bangladesh | Cluster-randomized effectiveness trial | N | Y | N | N |  |  |  |
| Legend: BEP=Balanced Energy-Protein; LNS=Lipid-based Nutrient Supplement; N=No; RCT=Randomized controlled trial; Y=Yes; | | | | | | | | |  |

**Supplemental Table 2**: Randomized trials that tested the efficacy of nutritious supplemental foods (BEP or LNS) in pregnant populations included in systematic reviews and meta-analyses

| **Study (N)** | **Location** | **Study Design** | **Participant Eligibility Criteria** | **Definition of Undernourishment** | **Interventions Description** | | **Nutritional Composition of Intervention** | | |  |
| --- | --- | --- | --- | --- | --- | --- | --- | --- | --- | --- |
|  |  |  |  |  | Food supplement provided to the control group (if any) | Food supplement in the intervention group | Calories | Protein | Fat |  |
| **BEP Studies** | | | | | | | | | |  |
| Atton 1990 (N=452) | England, UK | Quasi-RCT | Asian women ≤18 weeks of gestational age were included as well as women of other races if they were underweight (BMI <20 kg/m^2^; under 50 kg) at enrolment, or previous history of small babies, late miscarriages, or premature labor | BMI <20 kg/m^2^ and under 50 kg at enrollment | None given, participants allowed to follow the standard of care; except for Asian participants who also received calcium and vitamin D tablets | Flavored milk product packaged in a 200-ml Tetrabrick carton (with choice of flavors) | 407 kcal | 14.6 g | 5.85 g |  |
| Blackwell 1973 (N=294) | Taiwan | RCT | Well-nourished rural Taiwanese women with ‘marginal' diets, married, planned to have at least one more child, of low socioeconomic status, recruited in the last trimester of pregnancy | Low socioeconomic status based on lack of electric appliances in the home; women had a ‘marginal’ diet based on a preliminary food survey in 1965 in this area that estimated a daily energy intake of approximately 2000 kcal and protein intake ≤ 40 g for adult women | Chocolate flavored drink: first 4 years, 6 kcal/day and then 40 kcal/day as artificial sweetener replaced with sucrose (12 ounce can); MN added in the last year near completion of the study | Chocolate milk flavored energy drink + MN (12 ounce can) | 400 kcal | 20 g | 13.3 g |  |
| Campbell-Brown 1983 (N=2918) | Scotland, UK | Controlled Trial | Primiparous women at high risk of low birth weight delivery, at ~ 27 weeks gestation | Risk of low birthweight was based on meeting ≥ 2 of the following criteria: low maternal height, weight or weight-for-height at 20 weeks, or weight gain between 20 and 30 weeks, nutritional status in early life. Participants of this study were considered malnourished | None given | Three different supplement options were offered based on subjects’ preference: 0.5 pint of flavored milk drink, 1 pint of fresh milk, 75 g cheddar cheese | 300 kcal | 14.6 g |  |  |
| Ceesay 1997 (N=2082) | Gambia | Cluster- RCT | Gambian women from 28 rural villages with “chronically” marginal nutrition. The mean maternal BMI measured after delivery was 20.7 ± 2.3 kg/m^2^ in the control group and 21.3 ± 2.8 kg/m^2^ in the intervention group | Chronically undernourished women (twin bearers excluded), yielding 2047 singleton live births and 35 stillbirths. Undernutrition more pronounced from June to October (the ’hungry’ season involving low food supply and heavy agricultural work) than from November to May (the dry harvest season with adequate food supply and less strenuous work) | None given, antenatal care available - all women received IFA, based on their hemoglobin concentration | Two high energy groundnut biscuits containing roasted groundnuts, rice flour, sugar, and groundnut oil | 1016 kcal | 22 g | 56 g |  |
| Dwarkanath 2016 (N=24) | India | RCT | 24 Indian (Bangalore) pregnant women at < 13 weeks gestation with low BMI (≤18.5) | Low BMI (≤18.5) | None given; first trimester: 5 mg folic acid; second trimester - delivery: 0.5 mg folic acid, 45 mg iron, 1 g calcium were prescribed | Three small, round “granola-type” treats, called ladoos (made of crushed roasted peanuts, puffed rice, skimmed milk, clarified butter, and unrefined sugar) | 300 kcal | 15 g | - |  |
| Elwood 1981 (N=1251) | South Wales, UK | Pre/post design | Pregnant Welsh women from 2 small towns recruited at time of first reporting of pregnancy | None defined | None given | Free tokens to purchase milk (240 mL) for their families | 150 kcal | 8 g | - |  |
| Girija 1984 (N=20) | India | RCT | Indian women (20-33 yrs.) in the last trimester, 2-7 gravida, belonging to a low socioeconomic group (monthly family income < Rs. 700 (US $100)) | None defined | None given; IFA given to pregnant women via the national program and at times, food supplements are given during the last trimester | 50 g of sesame cake, 40 g jaggery, and 10 g oil | 417 kcal | 30 g | - |  |
| Huybregts 2009 (N=1296) | Burkina Faso | RCT | Pregnant women referred to the health center | None defined; ~ 13% of participants had BMI <18.5 kg/m^2^ and ~40% had hemoglobin <11.5 g/dL. BMI at entry for intervention group was 20.8 ± 2.2 kg/m^2^, and for control group was 21.0 ± 2.2 kg/m^2^ | None given; MMN supplement only | 72 g of a prenatal MMN-fortified spread consisting of 33% peanut butter, 32% soy flour, 15% vegetable oil, 20% sugar and an MMN at 1x RDA | 372.6 kcal | 14.7 g | Fat (g) 27.6  SFA (g) 8.1 MUFA (g) 12.1 PUFA (g) 7.3 omega-3 Fatty acids (g) 0.4 omega-6 Fatty acids (g) 7.0 |  |
| Kardjati 1988 (N=747) | Indonesia | RCT | Pregnant women from 3 rural villages from East Java region (an area known to be ’nutritionally vulnerable at 26-28 weeks’ gestation). Total mean ± SD pre-pregnancy BMI was 18.7 ± 2.0 kg/m^2^ | None defined; from previous nutrition surveys from the same area, it was known that habitual diets of pregnant women were mainly deficient in energy | Supplement containing 52 kcal energy and 6.2 g protein (‘low energy’); free antenatal care available | Supplement containing dry powder (50% fat, 10% casein, 40% glucose) | 465 kcal | 7.1 g | 18 g |  |
| Kaseb 2002 (N=53) | Iran | Sequentially randomized intervention study | Healthy Iranian women in the 4^th^ month of pregnancy attending the local health centers; all participants considered adequately nourished | None defined | None given; all women received prenatal care | Food supplementation: rice-milk porridge, lentils, pottage, cheese, yogurt, eggs, milk with bread | 400 kcal | 15 g | - |  |
| Mardones-Santander 1988 (N=1135) | Chile | Quasi-RCT | Low-income women were included if: > 18 years, parity 0–5, <20 weeks pregnant according to date of last menstrual period, non-smoking, non-alcoholic, and were underweight. Women with multiple pregnancies were excluded at delivery | Underweight defined based on weight for height at the first prenatal visit (at mean gestation of 14 weeks) < 95% of the standard at week 12 of gestation; all women of low-income | The ‘PUR group,’ received powdered milk (an isocaloric supplement) with MMN | ‘V-N group’ received a fortified formula milk (BEP supplement) with MMN; also, through the same program all women received 2 kg of rice monthly | PUR: 498 kcal V-N: 470 kcal | PUR: 27.9 g  V-N: 14.5 g | PUR:  Fat (g) 26  Milk fat (g) 26  Vegetable fat (including 5.25 g of linoleic acid);  V-N:  Fat (g) 21  Milk fat (g) 10.5  Vegetable fat (including 5.25 g of linoleic acid) 10.5 |  |
| Metcoff 1985 (N=900) | United States | pre/post design | Women selected through the low-income program, WIC, and attending the prenatal clinics at the Oklahoma Memorial Hospital | WIC inclusion criteria includes undernourishment and low socioeconomic status | None given | Monthly WIC vouchers for supplements of milk, egg, and cheese | 900-1000 kcal | 40-50 g | - |  |
| Mora 1978 (N=456) | Colombia | RCT | Low-income women from a Bogota slum in their first- or second-trimester | None defined; all of low-income | None given; uniform health care program available, which includes social, health, and child development measures | Supplement provided 60 g of dry skim milk, 150 g of enriched bread, 20 g of vegetable cooking oil, and a vitamin/mineral supplement | 856 kcal | 38 g | vegetable oil-20 g |  |
| Oaks 2014 (N=1320) | Ghana | RCT | Pregnant women who attended antenatal clinics at two selected hospitals and one polyclinic. They were invited for screening if aged 18y or above, and ≤ 20 week of gestation (determined by the antenatal clinics, mostly by fundal height) | None defined | None given; Control 1: IFA (60 mg iron, 400 μg folic acid)  Control 2: MMN tablet, 1–2 RDA of 18 vitamins and minerals (including 20 mg iron) | SQ LNS: spread made out of vegetable oil, powder milk, groundnut paste, sugar, and MMN; included the same micronutrients as the MMN group, plus another 4 minerals (Ca, P, K, and Mg) | 118 kcal | 2.6 g | 10 g |  |
| Prentice 1987 (N=197) | Gambia | Non-randomized intervention (pre/post design) | Women were enrolled as soon as pregnant. Low rainfall meant the harvests were rarely sufficient to last the entire year and a hungry season develops between July and September | None defined | None given; antenatal care – all women were prescribed daily iron (47 mg) and folate (500 g pteroyl glutamic acid) as FEFOL | Groundnut-based biscuits and a vitamin-fortified tea drink: maximum uptake of the supplement-3x65 g biscuits and 380 g tea in the dry season. 4x65 g biscuits and 380 g tea in the hungry season (July – October) | A) biscuits: 468 kcal;  B) 380 g fortified tea: 78 kcal | A) 17.4 g;  B) 2.9 g | A) 25.5 g; B) 1.6 g |  |
| Ross 1985 (N=127) | South Africa | RCT | Black South African, urban women < 20 weeks gestation | None defined | None given; Group 1: placebo Group 2: zinc (30-90 mg zinc gluconate daily); all women in the study had routine medical antenatal care | Group 3: high bulk supplement- beans and maize; Group 4: low bulk supplement-porridge with 100 g dry skimmed milk, maize, flour, vitamins, and minerals | High bulk: 776 kcal;  Low bulk: 700 kcal | Vegetable source:  high bulk: 36 g  low bulk: 8 g;  Animal source: high bulk: 0 g  low bulk: 36 g | - |  |
| Rush 1980 (N=814) | United States | RCT | Women selected from the New York City Municipal Hospital. Eligible women were black, English speaking, no more than 30 weeks’ gestation at interview | Weighed < 140 lbs. at conception and had at least one of the following: low pre-pregnancy weight (<110 lbs. at conception), low weight gain at the time of recruitment, at least one previous low-birth-weight infant, a history of protein intake of less than 50 g in 24 hrs. Preceding registration as calculated by the nutritionist from 24 hr quantitative dietary recall | None given; participants received a continuation of regular clinic care, which included standard multivitamin/mineral | Supplement (Supp): a 16-oz beverage (high protein-energy with multivitamin/mineral); | Supp: 470 kcal; | Supp: 40 g; | Supp: 8.6 g; |  |
|  |  |  |  |  |  | Complement (Comp): a 16-oz drink (balanced energy and protein with multivitamin/mineral) | Comp: 322 kcal | Comp: 6 g | Comp: 7.6 g |  |
| Tontisisirin 1986 (N=43) | Thailand | RCT | Non-smoking mothers (16-30 yrs.), all from a rural region, of the same socioeconomic class, selected from the local hospital, in the first or second pregnancy, all at 28 weeks (±2 wks) of gestation | None defined | None given | Group 1- Formula I: roast beans and sesame for 10-15 min, cook groundnuts for 15-20 mind and ground  Group 2- Formula V: dry cooked rice exposed to sunlight for 3-4 h or drying in an oven at 250 °C for 20 min, fry dried rice in the oil for 3 s, mixed with ground nuts and dried shrimp, sticky melting sugar stirred in | 350 kcal | 13 g | - |  |
| Viegas 1982 a) (N=153) | UK | RCT | Asian women from Birmingham, UK < 20 weeks’ gestation who appeared well-nourished based on their height and weight and same distribution of parity, abnormal obstetric history, and history of early bleeding in the current pregnancy | None defined | Group 1: Vi-control= half a bottle (369 mL) daily of flavored carbonated water containing iron (3 mg) and vitamin C (30 mg) | Group 2:  EnVit = Vi + flavored carbonated dietary protein energy supplement | 273 kcal | Group 3: 26 g | - |  |
|  |  |  |  |  |  | from 18 to 38 weeks.  Group 3: PrEnVit: Vi + containing 1/3 liquid glucose drink, chocolate flavored skim milk powder (26 g provided daily) |  |  |  |  |
| Viegas 1982 b) (N=130) | UK | RCT | Enrolled Asian women <20 weeks of gestation, in Birmingham who appeared well-nourished (based on height and weight) prior to pregnancy. Received iron (3 mg daily) and vitamin C (30 mg daily) from 18-20 weeks until 28 weeks. Later, 45 women selected by an inadequate increase in triceps skinfold thickness increment (<20 μm/week) during the second trimester | 45 of women were later considered “nutritionally at risk” based on inadequate increase in triceps skin folds (between 18 and 28 weeks) stratified at 28 weeks according to increase in triceps skinfold during second trimester (<20 μm/week) | None given; Group 1: Vi-control= a multivitamin sachet (Orovite7) | Group 2: EnVi= Vi + glucose syrup (Hycal) providing 425 kcal;  Group 3: PrEnVi= EnVi, but 10% of energy provided by protein in chocolate- flavored skimmed-milk powder (40 g/d) | Group 2 & 3: 425 kcal | Group 3: 40 g | - |  |
| **LNS Studies** | | | | | | | | | |  |
| Adu-Afarwuah 2015 (N=1320) | Ghana | RCT | Pregnant women who attended antenatal clinics at two selected hospitals and one polyclinic. They were invited for screening if aged 18y or above, and ≤ 20 week of gestation (determined by the antenatal clinics, mostly by fundal height) | None defined | None given; Control 1: IFA (60 mg iron, 400 μg folic acid)  Control 2: MMN tablet, 1–2 RDA of 18 vitamins and minerals (including 20 mg iron) | SQ LNS: spread made out of vegetable oil, powder milk, groundnut paste, sugar, and MMN;  LNS group had the same micronutrients as the MMN group, plus another 4 minerals (Ca, P, K, and Mg), as well as macronutrients | 118 kcal | 2.6 g | 10 g |  |
| Ashorn 2015 (N=1391) | Malawi | RCT | Pregnant women (>15 yrs. of age) in 1 public district hospital, 1 rural semiprivate hospital, and 1 rural public health center in Southern Malawi; ultrasound scan confirmed pregnancy of ≤ 20 weeks of gestation | None defined | None given; Control 1: IFA (60 mg iron, 400 μg folic acid)  Control 2: MMN tablet, 1–2 RDA of 18 vitamins and minerals (including 20 mg iron) | SQ LNS + MMN+ Ca, Phosphorus, K, Mg: same as Adu-Afarwuah 2015 | 118 kcal | 2.6 g | 10 g |  |
| Huybregts 2009 (N=1296) | Burkina Faso | RCT | Pregnant women from Burkina Faso where the prevalence of low birth weight is 16% and there are multiple MN deficiencies. Pregnant women were referred to the health center with no exclusion criteria other than those who planned to leave the study area within the next 2 years | None defined | None given; MMN: UNIMMAP composition | LQ LNS: fortified food supplement, which is a fortified spread consisting of 33% peanut butter, 32% soy flour, 15% vegetable oil, 20% sugar, and an MMN cocktail (meet RDA for women, equal to UNIMMAP) | 1.56 MJ (372 kcal) | 14.7 g | 27.6 g |  |
| Moore 2012 (N=875) | Gambia | RCT | Pregnant women (from 12 weeks gestation to term) 18-45 years from West Kiang region (rural subsistence farming community); ultrasound scan conformed pregnancies between 10-20 weeks at a clinic | None defined | None given; Control 1: IFA (60 mg iron, 400 μg folic acid)  Control 2: MMN tablet (15 MN for pregnancy and formulated by UNIMMAP) | Group 1: LQ LNS; Protein-energy LNS + IFA + Vitamin A, E, C, B1, B2, niacin, B6, B12, Zn, Cu, Se, and Iodine.  Group 2: LQ LNS; Protein-energy LNS + MMN | 746 kcal | 20.8 g | 52.6 g |  |
| Mridha 2016 (N=4011) | Bangladesh | Cluster-randomized effectiveness trial | Pregnant and lactating women who joined the Rang-Din Nutrition Study in a community health program in rural Bangladesh (area with one of the highest levels of poverty). Women were enrolled if ≤ 20 week of gestation; pregnancy was confirmed based on stopped menstruation, urine pregnancy test, and in some cases ultrasonogram report | None defined | None given; IFA: 60 mg iron, 400 μg folic acid | Maternal SQ LNS: same as Adu-Afarwuah 2015; also have child-only intervention groups, omitted for the purpose of this review | 118 kcal | 2.6 g | 10 g |  |
| Abbreviations: BEP=Balanced Energy-Protein Supplements; IFA=Iron and folic acid; LQ LNS=Large-quantity Lipid-based Nutrient Supplements; MMN=Multiple micronutrients; MUFA=Medium-chain Fatty Acid; PUFA=Poly-unsaturated Fatty Acid; RCT=Randomized controlled trial; RDA=Recommended Dietary Allowance; SFA=Short-chain Fatty Acid; SQ LNS=Small-quantity Lipid-based Nutrient Supplements; UNIMMAP= United Nations International Multiple Micronutrient Antenatal Preparation; WIC=Special Supplemental Nutrition Program for Women, Infants, and Children | | | | | | | | | |  |
|  |  |  |  |  |  |  |  |  |  |  |

**Supplemental Table 3**. Micronutrient composition of nutritious supplemental foods (BEP and LNS) included in systematic reviews and meta-analyses

| **Selected Micronutrients^a^** | **Vit A (μg RAE)^b^** | **Vit D (IU)** | **Vit E (mg)^c^** | **Vit B1 (mg)** | **Vit B2 (mg)** | **Vit B3 (mg)^d^** | **Vit B6 (mg)** | **Folic Acid (μg)** | **Vit B12 (μg)** | **Vit C (mg)** | **Iron (mg)** | **Calcium (mg)** | **Magnesium (mg)** | **Zinc (mg)** | **Copper (mg)** | **Iodine (μg)** | **Phosphorus (mg)** |  |
| --- | --- | --- | --- | --- | --- | --- | --- | --- | --- | --- | --- | --- | --- | --- | --- | --- | --- | --- |
| **BEP Studies** | | | | | | | | | | | | | | | | | |  |
| Atton 1990, INT | 37.5 | 20 | - | 0.1 | 0.2 | 1.5 | - | 20 | - | 3.8 | 0.8 | 141 | - | 1 | 0.1 | - | 109 |  |
| Blackwell 1973, INT | 750 | 200 | 3.4 | 0.8 | 0.9 | 10 | 0.8 | - | 1 | 37.5 | 6 | 500 | - | - | 0.5 | - | 400 |  |
| Ceesay 1997, INT | - | - | - | - | - | - | - | - | - | - | 1.8 | 47 | - | - | - | - | - |  |
| Dwarkanath 2016, INT | - | - | - | - | - | - | - | 500-5000^†^ | - | - | 45 | 1000 | - | - | - | - | - |  |
| Huybregts 2009, INT | 881 | 200 | 13 | 1.6 | 1.6 | 21 | 2 | 461 | 2.6 | 71 | 35 | 90 | - | 17 | 2.7 | 150 | - |  |
| Huybregts 2009, CN | 800 | 200 | 10 | 1.4 | 1.4 | 18 | 1.9 | 400 | 2.6 | 70 | 30 | - | - | 15 | 2 | 150 | - |  |
| Kardjati 1988, INT | 443 | - | - | 0.83 | 0.36 | - | - | - | - | - | 6.7 | 167 | - | - | - | - | - |  |
| Mardones-Santander 1988, INT (V-N) | 1100 | 432 | 2.1 | 0.3 | 0.8 | 3.3 | 0.7 | 400 | - | 340 | 43 | 860 | 130 | 6.5 | 0.5 | 60 | 660 |  |
| Mardones-Santander 1988, CN (PUR) | 270 | 12 | 0.4 | 0.3 | 0.8 | 0.8 | 0.5 | 50 | - | 10 | 0.5 | 1030 | 105 | 3.5 | 0.3 | 45 | 810 |  |
| Mora 1978, INT | 1807 | - | - | - | - | - | - | - | - | - | 18 | - | - | - | - | - | - |  |
| Prentice 1987, INT (Biscuits) | 0 | - | - | - | 0.2 | - | - | 500 | - | 0 | 47 | 180 | - | - | - | - | - |  |
| Prentice 1987, INT (Tea) | 340 | - | - | - | 0.2 | - | - | 500 | - | 10 | 47 | 180 | - | - | - | - | - |  |
| Prentice 1987, CN | - | - | - | - | - | - | - | 500 | - | - | 47 | - | - | - | - | - | - |  |
| Ross 1985, INT (HBS) | - | - | - | 0.3 | 0.3 | 4 | - | - | - | 40 | 9.3 | - | - | - | - | - | - |  |
| Ross 1985, INT (LBS) | 1200 | - | - | 0.7 | 1.9 | 2.2 | - | - | - | 40 | 2.8 | 1300 | - | - | - | - | - |  |
| Ross 1985, INT (Zinc gluconate) | - | - | - | - | - | - | - | - | - | - | - | - | - | 30-90 | - | - | - |  |
| Rush 1980, INT (Supplement) | 1800 | 400 | 20.1 | 3 | 15 | 15 | 2.5 | 350 | 8 | 60 | 60 | 1000 | 100 | 4 | 2 | 150 | - |  |
| Rush 1980, CN (Complement) | 1200 | 400 | - | 3 | 15 | 10 | 3 | 350 | 3 | 60 | 40 | 250 | 12 | 0.1 | 0.2 | 100 | - |  |
| Rush 1980, CN | 1200 | 400 | - | 3 | 2 | 10 | 3 | 350 | 3 | 60 | 117 | 250 | 0.2 | 0.1 | 0.2 | 100 | - |  |
| Viegas 1982 a) CN & INT | - | - | - | - | - |  | - | - | - | 30 | 3 | - | - | - | - | - | - |  |
| Viegas 1982 b) CN & INT | 750 | 100 | - | 1.4 | 1.7 | 18 | 2 | - | - | 60 | 3 | - | - | - | - | - | - |  |
| **LNS Studies** | | | | | | | | | | | | | | | | | |  |
| Adu-Afarwuah 2015, INT (LNS) | 800 | 400 | 20 | 2.8 | 2.8 | 36 | 3.8 | 400 | 5.2 | 100 | 20 | 280 | 65 | 30 | 4 | 250 | 190 |  |
| Adu-Afarwuah 2015, CN (MMN) | 800 | 400 | 20 | 2.8 | 2.8 | 36 | 3.8 | 400 | 5.2 | 100 | 20 | 0 | 0 | 30 | 4 | 250 | 0 |  |
| Adu-Afarwuah 2015, CN (IFA) | - | - | - | - | - | - | - | 400 | - | - | 60 | - | - | - | - | - | - |  |
| Ashorn 2015, INT (LNS) | Same as Adu-Afarwuah 2015 | | | | | | | | | | | | | | | | |  |
| Huybregts 2009, INT (LNS) | 881 | 200 | 13 | 1.6 | 1.6 | 21 | 2 | 461 | 2.6 | 71 | 35 | 90 | - | 17 | 2.7 | 150 | - |  |
| Huybregts 2009, CN (MMN) | 800 | 200 | 10 | 1.4 | 1.4 | 18 | 1.9 | 400 | 2.6 | 70 | 30 | - | - | 15 | 2 | 150 | - |  |
| Moore 2012, INT (LNS + MMN) | 1600 | 400 | 20 | 2.8 | 2.8 | 36 | 2.8 | 400 | 5.2 | 140 | 60 | - | - | 30 | 4 | 300 | - |  |
| Moore 2012, INT (LNS + IFA) | 2.9 | - | 4.2 | 0.3 | 0.5 | 1.4 | 0.2 | 400 | 0.1 | 2.3 | 60 | - | - | 3.3 | 1.1 | 2.6 | - |  |
| Moore 2012 CN (MMN) | 1600 | 400 | 20 | 2.8 | 2.8 | 36 | 2.8 | 400 | 5.2 | 140 | 60 | - | - | 30 | 4 | 300 | - |  |
| Moore 2012 CN (IFA) | - | - | - | - | - | - | - | 400 | - | - | 60 | - | - | - | - | - | - |  |
| Mridha 2017 | Same as Adu-Afarwuah 2015 | | | | | | | | | | | | | | | | |  |
| ^a^Additional micronutrient compositions of the supplements in each study: BEP studies: Atton 1990, CN: indicated that antenatal care was available and calcium and vitamin D were given to Asian participants only; Blackwell 1973, INT: 0.6 mg fiber, 1.0 mg manganese, 900.0 mg potassium, 200.0 mg sodium; CN: MMN were added in the last year near completion of the study but their composition was not disclosed; Ceesay 1997, CN: antenatal care available, all women received IFA (content was not disclosed), based on their hemoglobin concentration; Dwarkanath 2016, INT: ^†^1^st^ trimester 5000.0 ug/day folic acid, 2^nd^ trimester 500.0 ug/day folic acid; CN: consumed their habitual diet, a ladoo was consumed after each meal; Huybregts 2009, INT (BEP/LNS): 0.01 mg fiber, 0.5 mg folate, 0.7 mg selenium; Kardjati 1988, CN: free antenatal care available (composition not disclosed); Mora 1978, CN: all groups received a uniform health care program and the same social, health, and child development measurements throughout the study period; Ross 1985, CN was placebo and did not include micronutrients; Rush 1980, all groups also received 78.0 mg iron and 1.0 mg pantothenic acid; LNS studies: Adu-Afarwuah 2015, INT (LNS): 200.0 mg potassium; The following studies were not included in the table because they did not indicate iron prescription or antenatal care availability (1–4). ^b^As retinol equivalents (RE). In preformed vitamin A, RE has the same conversion factors as retinol activity equivalents (RAE). 1 RAE= 1 μg retinol, 12 μg β-carotene, or 24 μg β-cryptoxanthin. ^c^As α-tocopherol, α-tocopherol includes RRR- α-tocopherol, the only form of α-tocopherol that occurs naturally in foods and the 2R-stereoisomeric forms of α-tocopherol (RRR-, RSR-, RRS-, and RSS- α-tocopherol) that occur in fortified foods and supplements. It does not include the 2s-stereoisomeric forms of α-tocopherol (2SRR-, SSR-, SRS and SSS- α-Tocopherol), also found in fortified foods and supplements. ^d^As niacin equivalents (NE). 1 mg of niacin=60 mg of tryptophan; 0-6 months=performed niacin (not NE). ^*^4 mg of ferrous fumarate fine powder + 2.5 mg iron-sodium EDTA. Abbreviations: BEP=Balanced-Energy Protein Supplements; CN=Control; FFS=Fortified food supplement; HBS=High bulk supplement; IFA=Iron and folic acid; INT=Intervention; IU=International Units; LBS=Low bulk supplement; LNS=Lipid-based nutrient supplements; mg=milligrams; μg=micrograms; MMN=Multiple micronutrient; NE= Niacin equivalents; PUR=Powdered milk, RAE=Retinol activity equivalents; RE=Retinol equivalents; V-N=Milk-based fortified product; Vit=Vitamin | | | | | | | | | | | | | | | | | |  |
|  |  |  |  |  |  |  |  |  |  |  |  |  |  |  |  |  |  |  |
|  |  |  |  |  |  |  |  |  |  |  |  |  |  |  |  |  |  |  |
|  |  |  |  |  |  |  |  |  |  |  |  |  |  |  |  |  |  |  |
|  |  |  |  |  |  |  |  |  |  |  |  |  |  |  |  |  |  |  |
|  |  |  |  |  |  |  |  |  |  |  |  |  |  |  |  |  |  |  |
|  |  |  |  |  |  |  |  |  |  |  |  |  |  |  |  |  |  |  |
|  |  |  |  |  |  |  |  |  |  |  |  |  |  |  |  |  |  |  |
|  |  |  |  |  |  |  |  |  |  |  |  |  |  |  |  |  |  |  |

| **Supplemental Table 4**: Outcomes measured in trials of BEP and LNS supplementation during pregnancy from ongoing/recently completed studies and those included in systematic reviews and meta-analyses | |
| --- | --- |
| **Study citation** | **Study outcomes** |
| **BEP/LNS from ongoing/recently completed studies** |  |
| ENAT | Newborn weight and length (< 72 hours at birth), length of gestation, spontaneous delivery (<37 weeks gestation), livebirths (<37 weeks gestation), SGA, LBW, stillbirth, newborn head circumference, weight-for-age, length-for-age, head circumference-for-age z scores (<72 hours at birth), gestational weight gain, anemia (third trimester) |
| MISAME-III | SGA, LGA, LBW, preterm birth, gestational duration, birth weight, birth length, Rohrer’s ponderal index at birth, head circumference, thoracic circumference, arm circumference, fetal loss, stillbirth |
| MINT | SGA, length-for-age z-score at 6 months, short-for-GA (<10^th^ percentile), SGA (<3^ed^ percentile), short-for-GA (<3^ed^ percentile), birth weight, length, GA, LBW, livebirths (<37 weeks gestation), weight-for-age, length-for-age, weight-for-length z scores (at 6 and 12 months), breastmilk composition, gestational weight gain, gut microbiome diversity in mother and infant, maternal weight and BMI |
| MumtaPW | Birth weight, length, head circumference, MUAC (< 72 hours at birth), weight velocity, length velocity, length-for-age, weight-for-age, and weight-for-length z scores from birth – 11 months, maternal height, weight, MUAC, and BMI, neurodevelopment assessment, early learning and motor ability, neurological examination, Global Scale for Early Development, brain morphology (all in infant), maternal depression (antenatal and postpartum period), maternal blood biomarkers (hemoglobin, ferritin, vitamin D, niacin), maternal urine (choline), maternal and infant stool, cord blood, breast milk |
| WINGS | Preterm birth, LBW, SGA, stunting (24 months) and if these outcomes differ by maternal status (<150cm or ≥150cm) |
| Niger (LNS intervention) | Length for age, weight for age, weight for length z-scores at 24 months, growth trajectories of anthropometric z scores from 6-8 weeks to 24 months |
| **BEP studies** |  |
| Atton 1990 | Maternal triceps skinfold thickness throughout pregnancy, maternal weight, infant anthropometry at birth |
| Blackwell 1973 | Gestational weight gain, preterm birth, birthweight, SGA, length, head circumference, and IQ at age 5 |
| Briley 2002 | LBW |
| Ceesay 1997 | Gestational weight gain, GA, birthweight, birth length, head circumference, stillbirth, and neonatal death |
| Campbell-Brown 1983 | Dietary assessment using 7-day food recalls, urinary nitrogen excretion during pregnancy, maternal weight gain, length of gestation, infant anthropometry at birth. |
| Dwarkanath 2016 | Fetal loss, maternal weight gain, GA, birth weight, birth length, premature deliveries, SGA, LBW |
| Elwood 1981 | GA, preterm birth, birthweight, LBW, length, and head circumference |
| Girija 1984 | Gestational weight gain, birthweight, length, head circumference, breast milk output, and weight,  length, and head circumference, through 3 months of age |
| Hunt 1976 | Protein and energy intakes |
| Jahan 2014 | Gestational weight gain (7 to 9 months), birth weight of newborn, LBW, the rate of initiation of breastfeeding  within 1 hour after birth |
| Huybregts 2009 | Anthropometric measures at birth, LBW, infant born SGA, LGA, GA, preterm |
| Kardjati 1988 | Gestational weight gain, birthweight, and breast milk output |
| Kafatos 1989 | Energy and protein intake, serum vitamin and mineral levels, gestational weight gain, birthweight,  birth length and head circumference, GA, LBW, infant born SGA, preterm birth, stillbirth, and neonatal death |
| Kaseb 2002 | Maternal weight and weight gain, 24-hour food recalls, newborn weight and height, growth for the first four months |
| Mardones-Santander et al., 1983 | Maternal anthropometry, weight gain, birth outcomes, neonatal anthropometry, gestational duration |
| Metcoff 1985 | Maternal weight at 19- and 26-weeks gestation, birth anthropometry, low birthweight, gestational age, several nutrients measured in blood |
| Mora 1978 | Pre-eclampsia, GA, preterm birth, birthweight, LBW, stillbirth, perinatal mortality, neonatal mortality |
| Oaks 2014 | Gestational duration, preterm birth, infant weight (weight-for-age z-score), length (length-for-age z-score), and head circumference (head circumference-for-age z-score) |
| Prentice 1987 | Birthweight, GA, gestational weight gain, health care services, home food intake measurements |
| Ross 1985 | Gestational weight gain (after 20 weeks), GA, and birthweight |
| Rush 1980 | Gestational weight gain, GA, preterm birth, infant born SGA, birthweight, LBW, stillbirth, neonatal mortality, and weight, length, head circumference, and Bayley scores at 1 year |
| Sweeney 1985 | Protein and energy intake, gestational weight gain, birthweight, and GA |
| Tontisirin 1986 | Maternal weight, triceps skin fold thickness, MUAC, 24-hour recall, blood hematocrit, newborn anthropometry |
| Viegas 1982 a) | Gestational weight gain, GA, birthweight, length, and head circumference, placental weight, and maternal skin folds |
| Viegas 1982 b) | Gestational weight gain, birthweight, placental weight, maternal skin folds, and arm circumference |
| **LNS studies** |  |
| Adu-Afarwuah 2015 | Infants’ birth length, birth length-for-age, birth weight, head circumference, weight-for-age, head circumference-for-age, BMI-for-age, GA at delivery, preterm delivery, LBW, low birth length, SGA, birth weight, head and mid-upper arm circumferences |
| Ashorn 2015 | Birth weight and newborn length, newborn weight, head and arm circumference, pregnancy duration |
| Huybregts 2009 | Birth weight, birth length, Rohrer’s ponderal index, LBW, SGA, LGA, thoracic circumference, head circumference, and mid upper arm circumference at birth, placental weight, preterm births, the percentage of miscarriages, stillbirths, and perinatal deaths |
| Moore 2012 | Infant immunity development, LBW |
| Mridha 2017 | Birth weight and length, GA, birth head circumference and z score, BMI-for-age z score, MUAC at birth, LBW, newborn stunting, preterm delivery, SGA |
| Abbreviations: BEP=Balanced Energy-Protein Supplements; BMI=Body mass index; ENAT=Enhancing Nutrition and Antenatal Infection Treatment for Maternal and Child Health; GA= Gestational Age; IQ=Intelligence Quotient; LBW=Low birth weight; LGA=Large-for-gestational age; LNS=Lipid-based Nutrient Supplement; MINT=Mothers and Infants Nutrition Trial; MISAME III=MIcronutriments pour la SAnté de la Mère et de l’Enfant; MMF-BEP=Micronutrient Fortified Balanced Energy-Protein Supplements; MUAC=Mid-Upper Arm Circumference; MUMTA PW=Mumta Pregnant Women Trial; SGA= Small-for-gestational age; WINGS=Women and Infants Integrated Interventions for Growth Study | |
